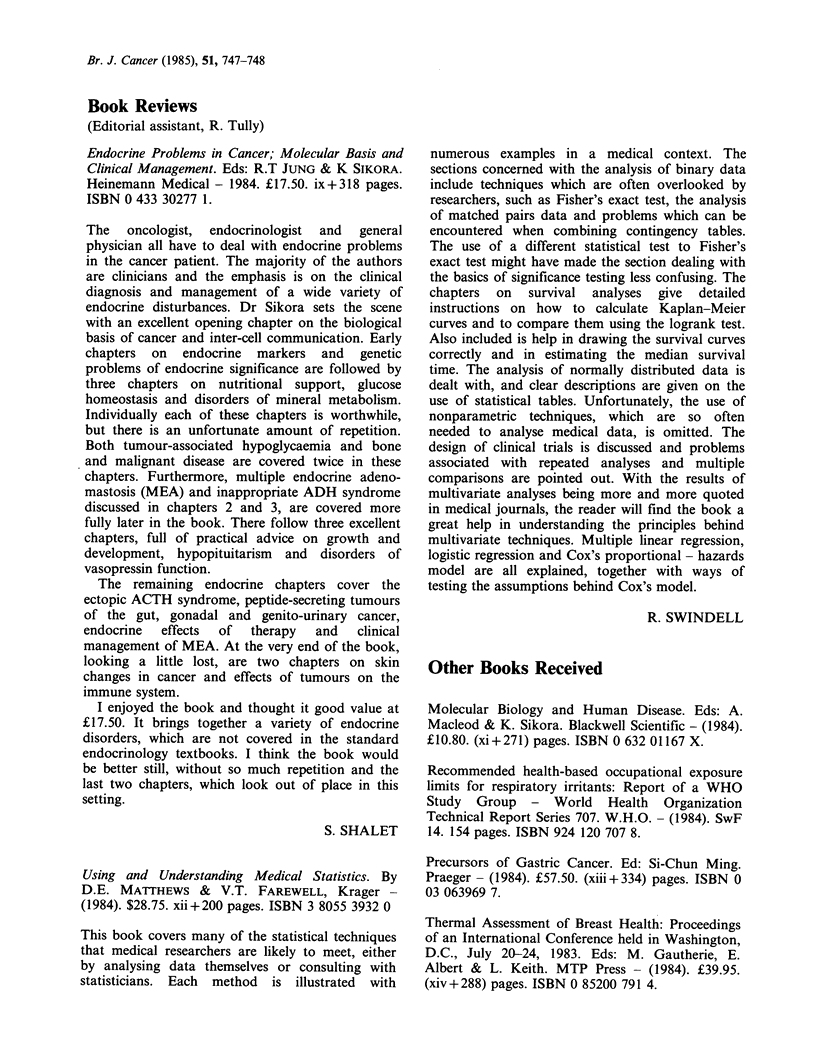# Using and Understanding Medical Statistics

**Published:** 1985-05

**Authors:** R. Swindell


*Using and Understanding Medical Statistics*. By D.E. MATTHEWS & V.T. FAREWELL, Krager – (1984). $28.75. xii+200 pages. ISBN 3 8055 3932 0

This book covers many of the statistical techniques that medical researchers are likely to meet, either by analysing data themselves or consulting with statisticians. Each method is illustrated with numerous examples in a medical context. The sections concerned with the analysis of binary data include techniques which are often overlooked by researchers, such as Fisher's exact test, the analysis of matched pairs data and problems which can be encountered when combining contingency tables. The use of a different statistical test to Fisher's exact test might have made the section dealing with the basics of significance testing less confusing. The chapters on survival analyses give detailed instructions on how to calculate Kaplan–Meier curves and to compare them using the logrank test. Also included is help in drawing the survival curves correctly and in estimating the median survival time. The analysis of normally distributed data is dealt with, and clear descriptions are given on the use of statistical tables. Unfortunately, the use of nonparametric techniques, which are so often needed to analyse medical data, is omitted. The design of clinical trials is discussed and problems associated with repeated analyses and multiple comparisons are pointed out. With the results of multivariate analyses being more and more quoted in medical journals, the reader will find the book a great help in understanding the principles behind multivariate techniques. Multiple linear regression, logistic regression and Cox's proportional – hazards model are all explained, together with ways of testing the assumptions behind Cox's model.

R. SWINDELL